# The Value Equation: Three complementary propositions for reconciling fidelity and adaptation in evidence-based practice implementation

**DOI:** 10.1186/s12913-019-4668-y

**Published:** 2019-11-21

**Authors:** Ulrica von Thiele Schwarz, Gregory A. Aarons, Henna Hasson

**Affiliations:** 1 School of Health, Care and Social Welfare, Mälardalen University, Box 883, 721 23 Västerås, Sweden; 2 Medical Management Centre, LIME, Karolinska Institutet, 171 77 Stockholm, Sweden; 3 Child and Adolescent Services Research Center, 3665 Kearny Villya Rd, Suie 200N, San Diego, CA 92123 USA; 4 Department of Psychiatry, University of California San Diego, 9500 Gilman Drive (0812), La Jolla, San Diego, CA 92093-0812 USA; 5 UC San Diego Dissemination and Implementation Science Center (UCSD-DISC), 9500 Gilman Drive (0990), La Jolla, CA 92093-0990 USA; 6 Unit for Implementation and Evaluation, Center for Epidemiology and Community Medicine, Stockholm County Council, Stockholm, Sweden

**Keywords:** Adherence, Modifications, Fidelity, Evaluation, Sustainability theory, Theory, Sustainment, Validity

## Abstract

**Background:**

There has long been debate about the balance between fidelity to evidence-based interventions (EBIs) and the need for adaptation for specific contexts or particular patients. The debate is relevant to virtually all clinical areas. This paper synthesises arguments from both fidelity and adaptation perspectives to provide a comprehensive understanding of the challenges involved, and proposes a theoretical and practical approach for how fidelity and adaptation can optimally be managed.

**Discussion:**

There are convincing arguments in support of both fidelity and adaptations, representing the perspectives of intervention developers and internal validity on the one hand and users and external validity on the other. Instead of characterizing fidelity and adaptation as mutually exclusive, we propose that they may better be conceptualized as complimentary, representing two synergistic perspectives that can increase the relevance of research, and provide a practical way to approach the goal of optimizing patient outcomes. The theoretical approach proposed, the “Value Equation,” provides a method for reconciling the fidelity and adaptation debate by putting it in relation to the value (V) that is produced. The equation involves three terms: intervention (IN), context (C), and implementation strategies (IS). Fidelity and adaptation determine how these terms are balanced and, in turn, the end product – the value it produces for patients, providers, organizations, and systems. The Value Equation summarizes three central propositions: 1) The end product of implementation efforts should emphasize overall value rather than only the intervention effects, 2) implementation strategies can be construed as a method to create fit between EBIs and context, and 3) transparency is vital; not only for the intervention but for all of the four terms of the equation.

**Summary:**

There are merits to arguments for both fidelity and adaptation. We propose a theoretical approach, a Value Equation, to reconciling the fidelity and adaptation debate. Although there are complexities in the equation and the propositions, we suggest that the Value Equation be used in developing and testing hypotheses that can help implementation science move toward a more granular understanding of the roles of fidelity and adaptation in the implementation process, and ultimately sustainability of practices that provide value to stakeholders.

## Background

Implementation science is defined as the scientific study of methods to promote the uptake of research findings into routine healthcare in clinical, organizational, or policy contexts [1]. The goal is to close the gap between what has been shown to be effective in rigorous trials (i.e., evidence-based interventions [EBIs], such as diagnostic tools and treatments) and what is done in clinical practice, so that patient and population health is improved.

If patients and ultimately populations are going to benefit from the best available evidence, fidelity (also denoted as adherence or treatment integrity), defined as the degree to which an intervention is carried out as it was described and originally tested and/or as the developer intended [2–8], is important in all steps of the research-to-practice pathway. During efficacy and effectiveness trials, when the main purpose is to separate effects of the intervention from other factors, high fidelity ensures that it is the intervention, not other factors, that produces the effect. In implementation studies, EBI fidelity is often a primary implementation outcome for determining if the methods used to promote uptake were successful [9]. In routine care, the degree to which EBIs are delivered as originally designed ultimately determines if patients and populations indeed receive the interventions that correspond with the best available evidence [2, 5, 10]. Overall, this makes understanding fidelity a central issue for implementation science.

However, throughout all the steps of the research-to-practice pathway, there are forces that pull away from fidelity. This has drawn increased attention to the role of adaptations, defined as the changes made to an intervention based on deliberate considerations to increase fit with patient or contextual factors at the system, organization, team, and individual clinician level [11–15]. *Deliberate* distinguishes adaptations from drift [16]. The central role of adaptations in implementation is increasingly being acknowledged, as evident from recent special issues [17] and themes in recent scientific meetings such as the US National Institutes of Health and Academy Health 9th Annual Conference on the Science of Dissemination and Implementation [10] and the 2018 Nordic Implementation Conference [18]. Yet, although there is global concern about fidelity-adaptation questions, the related conceptual and methodological issues are far from resolved.

Starting from routine care, there is ample evidence illustrating how adaptation of EBIs is the rule rather than the exception when used in real-world practice [12, 19, 20]. Influences on multiple levels, from the system, organization, provider, and patient, can all influence the degree to which EBIs might require adaptation [14, 21]. For example, reasons for adaptation can include system- and organization-level concerns including workforce readiness, organizational context, and the cost of purchasing EBIs from intervention developers and purveyors. In line with this, it has been noted that adaptability is an important factor that implementation strategies should address and that adaptation is likely to be needed to promote uptake [22]. This follows Everett Rogers’ seminal research stipulating that an innovation (e.g., an EBI) almost always will be reshaped to fit the organization or context in which it will be used [23]. In a concurrent development, research about cultural adaptations has also highlighted the need to tailor interventions based on the culture of the target populations [24], as well as the need to increase our understanding of cultural influences on implementation strategies and outcomes [25].

Recently, there has also been more discussion about the role that adaptations play earlier along the research-to-practice pathway [26, 27]. This includes questioning the assumption that fidelity automatically maximizes effectiveness [28]. It also includes showing that adaptation happens not only when EBIs are used in practice but also during trials, indicating that intervention researchers also needs to attend to issues related to fidelity and adaptation [27]. There have been a number of efforts to improve the reporting of fidelity and adaptation (e.g., [12, 13, 29–31]) (see also Roscoe et al., (2019) [32] for a comparison of four classification taxonomies), but to date, neither adaptations nor the intervention as planned (i.e., fidelity), are sufficiently described or documented in effectiveness trials [33–36]. This leaves a gap in understanding the full scope of the fidelity and adaptation dilemma earlier in the research-to-practice pathway. Thus, fidelity and adaptation are concepts that implementation science by necessity needs to acknowledge and address. Yet, this is prevented by the plethora of terms used, and the lack of clarity as to how the constructs can be conceptually organized. In Table 1, we propose a taxonomy that further refines the definitions of fidelity and adaptation, according to subcomponents and dimensions to which fidelity and adaptation can refer. This may aid in identifying relevant constructs for assessment and measurement.

**Table 1:** Definitions of subcomponents that represent dimensions suggested in the literature that fidelity and adaptation can refer to^a^

Sub-components	Characteristics of the intervention			Characteristics of the context of delivery	
	Content of intervention *What intervention activities and processes are performed?* [4, 37]	Intervention delivery *How is the intervention delivered?* [2, 37]	Format *In which format and through which channels is the intervention delivered?* [13, 24]	Conditions of the delivery context *What are the precise conditions related to the system, organization and providers under which the intervention is delivered?* [38]	*What is the target population specifics?* [7]
Suggested dimensions to consider	• The intervention *core components,* the specific active ingredients that make an EBI effective [7], *fidelity consistent* components [39] • The logic or theory that explains how the intervention is intended to work (i.e. the interventions’ *deep structure* [40]*, intervention strength* [38] and *program model* [7]; its *function* [41]) • Components that are part of the intervention but not central for producing the outcomes (*surface structure*) [40] or *adaptable* *periphery* • Components, not part of the intervention: *fidelity consistent* [5, 7, 39], or *fidelity inconsistent* [39] • Components that make the intervention uniquely distinguishable (program differentiation) [2]	• Number, length and frequency of sessions, • Density: how the intervention is spaced out in time; the intensity of the intervention [28] • The quality of delivery [2, 4] • What the treatment provider plans and *believes* is delivered (*dose delivered)* • What the recipient perceives that they have *received* (*dose received)* [42] • The timing; when the various parts of the intervention is delivered in relation to the other parts [43]	• Format of delivery, e.g. one-to-one or group • Channels of delivery, e.g. phone, internet or face-to-face • Location of delivery, e.g. school, non-profit organization or church	• Interventionist specifics (e.g. who is delivering the intervention; their training, competence level; personal attributes and skills) • Setting, e.g. primary care, hospital, community-based, workplace-based [13] • Organizational factors, e.g. climate, leadership, mandate, history [44] • System characteristics, e.g. reimbursement models, contracts, laws, policies, regulations, political climate [44]	• The health conditions that the intervention targets • Cultural characteristics • Age groups (e.g., children, youth, adults) • Patient’s own health goals and specific needs • Comorbidities

^a^Fidelity and adaptation sometimes refer to implementation strategies rather than interventions, that is, to what extent the strategies chosen to facilitate the use of the intervention is adhered to or adapted. The focus of the current paper is on fidelity to and adaptations of interventions

The issue of fidelity and adaptation has been controversial for decades (e.g., [45]). This debate deals with the longstanding tensions between achieving internal and external validity [46]. Whereas some scholars emphasize the importance of drawing valid conclusions about the effects of an intervention, thereby prioritizing internal validity, others highlight the need for interventions to fit and function in the daily operations of different systems and organizations, thus highlighting the virtue of external validity. However, there has been little theoretical development that can guide how fidelity and adaptations should be managed and documented across the research-to-practice pathway and how this is related to implementation science [21]. The debate and the research on fidelity and adaptations has been split and fragmented across multiple fields and journals representing various clinical fields, disciplines, and/or settings in which EBIs are implemented. With some noticeable exceptions (e.g., [13, 28]), the debate has taken place in parallel silos, and there is currently a lack of overview over the main arguments for fidelity on one side and adaptations on the other. This hampers a more comprehensive understanding needed to move toward a theoretical approach for how the fidelity and adaptation debate can be reconciled. This paper aims to synthesise the main arguments for fidelity and adaptation and, based on that, propose a theoretical and applied approach for how adaptation and fidelity can optimally be managed.

### Five reasons fidelity is vital – and five reasons adaptations are also vital

As outlined above, the logic of the research-to-practice pathway stipulates that EBIs should be used as they were described and intended to be provided. This approach implies that fidelity to the intervention is central and any deviations problematic. However, there are also strong arguments for why adaptations are needed. Table 2 summarizes the more pervasive reasons and justifications found in the literature for fidelity and adaptations, respectively.

**Table 2:** Arguments for Fidelity and Adaptation

Argument	Fidelity …	Adaptation …
1	… is vital for drawing valid conclusions by:	… improves intervention–context fit by:
	- increasing internal validity by the transparent and adherent use of EBIs [35] - separating implementation failure from theory failure, i.e., distinguishing between lack of effects due to insufficient *implementation* from lack of effects of an ineffective *intervention* [47] - avoiding type-III errors: concluding that an intervention is not effective when it actually was poorly implemented [48]	- ensuring that EBIs can be implemented and used in/for systems, organizations, providers, or patients that is different than the one in which the EBI was originally tested [49] - increasing the acceptability, feasibility, and applicability of an EBI to a given context [29] - increasing practical and/or value fit (philosophical and cultural) [12], e.g., creating fit with practical circumstances by changing from individual treatment to group format to align with funding contract [50]
2	… makes accumulation of knowledge possible by:	… balances different outcomes by:
	- making replication possible by ensuring the intervention remains the same across studies, thereby distinguishing between random and robust results [35, 51] - allowing results from multiple studies to be synthesised in systematic reviews and meta-analyses	- focusing on a broader spectrum of objectives, e.g., not only the specific clinical outcome an EBIs is evaluated against (e.g., symptom reduction, improved functioning) but also outcomes on other levels (patient, provider, organization and system) such as reach, relevance, costs [29, 49, 52] - focusing on optimizing benefits over time rather than focusing on sustained delivery (i.e., sustainment) [14, 28, 53, 54]
3	... assures EBI effectiveness by:	... assures EBI effectiveness by:
	- relying on studies, across different types of interventions and settings, showing that high fidelity can improve outcomes (e.g., [27, 37, 55–58] (at least in comparison to drift)	- relying on studies, across different types of interventions and settings, showing that adaptations can improve outcomes, e.g., [20, 59] (at least when there is a large variation in client and provider characteristics)
4	... provides transparency and confidence by:	... is necessary to address multiple diagnoses by:
	- ensuring that users, patients and their families, care providers, and funders (health systems, governments, insurers, and foundations) gets what they are promised [47, 60]: ... that patients know that the EBI offered is also the EBI delivered, facilitating informed choices ... that subsequent providers can deliver appropriate care; trusting that the treatment as documented in clinical records is also the treatment delivered ... that funders get what they are paying for ... that systems allow fair comparison between organizations in a competitive market, and fair benchmarking of treatment outcomes	- acknowledging that comorbidity is the rule rather than the exception in clinical practice, and that most EBIs have only been indicated (shown to be effective) for a very limited group of patients, primarily without comorbidities [61] - allowing “indication shift” to be able to use EBIs for groups for which evidence is lacking
5	... provides equal care and reduces disparities by:	... optimise the benefit for each patient by:
	- decreasing unwanted variation between providers, organizations, geographical regions, and different target groups or individuals, e.g., between men and women [60] - ensuring that decisions about adoption and use are made systematically, reducing the risk of gender and cultural biases	- translating mean effects into what is best for each individual in the group [62] - taking individual patient variability into account by detecting the individuals that are likely to improve less (i.e., the tails of the distribution of effects), consistent with the personalized medicine movement [63]

## Discussion

There are valid and reasonable arguments in support of fidelity, and there are valid and reasonable arguments in support of adaptation. However, many of the arguments seem to be contradictory and sometimes mutually exclusive. Much of the debate over the years has taken one or the other position, but the possibility that adaptation and fidelity can coexist has also been raised. These suggestions note that they can co-exist as long as the EBI core components are adhered to (e.g., [11, 24, 30]), and, more recently, that adaptation can improve fidelity by ensuring adherence to the key principles or elements underlying the EBI [64, 65]. Recent advancements in the conceptualization, measurement and documentation of adaptations have moved the field forward by aiding the empirical exploration of the relationship between fidelity, adaptations and outcomes (e.g., [14, 29, 31]).

Yet, with some noticeable exceptions (e.g., Chambers and Norton’s “Adaptome” model [26]), there have been few attempts at making theoretical propositions that address how fidelity and adaptation can be reconciled. In the following, we deconstruct the arguments for fidelity and adaptation to get at underlying assumptions, and then make three propositions that reconcile fidelity and adaptation. The propositions and equation terms are summarized in the Value Equation, as shown in Table 3. The Value Equation states that the optimal value (V) is a product of the intervention (IN), the nature of the context (C) in which the intervention is being implemented, and the implementation strategies (IS). The Value Equation (V = IN * C * IS) terms are described in detail below.

**Table 3:** The Value Equation: V = IN * C * IS

Terms	Specification	
Value (V)	Vs	Value and fit of intervention in the system context
	Vo	Value and fit of intervention in organizational context
	Vpr	Value and fit of the intervention for the provider
	Vpt	Value and fit of the intervention for the patient
Intervention (IN)	INf	Extent to which the intervention is carried out as it was described (fidelity)
	INfc	Fidelity-consistent adaptations
	INfi	Fidelity-inconsistent adaptations
Context (C)	Cs	System context
	Co	Organizational context
	Cpr	Provider context
	Cpt	Patient context
Implementation Strategy (IS)	ISc	Implementation strategy optimizing the context
	ISi	Implementation strategy optimizing the intervention

### Building the value equation

Table 3 summarizes elements of the Value Equation. Written as a simple mathematical equation, its starting point is an assumption that it is (only) the EBI that produces the effect:

$$ Intervention\ (IN)= Effect\ \left(E\ \right). $$

Implicit here is that by adhering to the intervention as it was designed, the 1) effect is maximized; 2) it is clear what is being delivered; 3) there is little unwanted EBI variation between organizations, professionals, and patients; and 4) it is possible to accumulate knowledge across studies. Nevertheless, as described previously, adaptation happens. Thus, there is a need to specify the intervention as the extent to which the intervention was carried out as it was described (fidelity) (INf), as well as fidelity-consistent (INfc) and fidelity-inconsistent (INfi) adaptations [39] (see Table 3).

As the EBI moves along the research-to-practice pathway, the influence of contextual factors is increasingly recognizable. Thus, a second term is added to the equation: Context (C).

$$ IN\ast C=E. $$

Because many implementations take place in complex systems including influences on system, organization, provider, and patient levels, context needs to be further specified. Thus, we suggest that context be delineated as system context (Cs), organizational context (Co), provider context (e.g., professional discipline, training, attitudes toward the intervention) (Cpr), and patient context (e.g., target group) (Cpt).

The Value Equation proposes that by acknowledging that context is indeed a term in the equation, the effects of intervention, by necessity, need to be understood in relation to the context in which it is implemented. For example, even in efficacy trials, there are contextual factors that will influence the outcome (e.g., highly trained staff delivering the intervention, urban settings). Thus, an EBI is not effective in isolation; it is more or less effective for a certain group, in certain settings, and under certain conditions. When the EBI is used beyond that, the context term changes, and so does the expected effect. High fidelity may increase effects in certain contexts, and adaptation in others. The optimal answers lie in the configuration of both terms in the equation.

### Implementation strategies create intervention–context fit

Implementation strategies are systematic processes to adopt and integrate EBIs into clinical care [22]. Implementation strategies can be simple (e.g., clinical reminders) or complex and multicomponent (e.g., training + coaching + audit and feedback) and varies with EBIs and contexts. We build on this notion to derive our first proposition: that implementation strategies are ways to create fit (i.e., appropriateness [9]) between an intervention and a specific context. We add a third term to the equation: Implementation Strategy (IS).

$$ IN\ast C\ast IS=E $$

We argue that implementation strategies can optimize the effect of interventions in two ways: 1) by optimizing the outer system or inner organizational context so that it fits the intervention (ISc) [44], or 2) by optimizing the intervention so that it fits the context (ISi) (Table 3). Thus, in the first case, implementation strategies are concerned with increasing *fidelity* by enabling appropriate changes in the context (e.g., by increasing competence among staff and/or create opportunities for the target behaviours through environmental restructuring such as changing the reimbursement system to allow clinicians needed time, etc. [66, 67]). In the second case, the implementation strategies promote *adaptations* to achieve fit (e.g., remove components because they are perceived as culturally inappropriate, or tailor based on patient preferences [12, 68]).

This proposition builds on the first argument for adaptation, stating that intervention–context fit is a necessary condition for implementation, but also invokes Elliott and Mihalic’s (2004) [69] notion that the need for intervention–context fit does not necessarily mean adaptation of the intervention; it may as well mean adaptation of the context to facilitate fidelity to the intervention. Thus, we build on previous work suggesting that adaptation and fidelity can co-exist (e.g., [11, 24, 30]), and add to that by explicitly proposing implementation strategies as the activities that optimize fit and reconcile fidelity and adaptation, whether those are concerned with modifying the intervention or the context, or both intervention and context.

The proposition to view implementation strategies as ways to create fit between an intervention and a specific context opens up new innovative approaches to choosing and matching implementation strategies, which has proven to be challenging [70]. The proposition aligns with recent suggestions to use user-centred design principles and community-academic partnerships for the purpose of creating fit between interventions and context, by engaging intervention developers and/or implementers and practitioners in a collaborative redesign process [71–73]. The value equation can aid this process by explicating which strategies are used, and why (if it is for the purpose of achieving fit by changing the context, or the intervention), and to what effect.

### Moving from effect to multilevel value proposition

A compelling argument for both fidelity and adaptation is the potential for increase in the effectiveness and public health impact of an intervention. Here, we make our second proposition by proposing an intentional shift from focusing on the *effect* of an intervention to focusing on the *value* (V) it creates, making a final adjustment to the equation by exchanging effect for value. Expressed mathematically, the complete Value Equation becomes the following:

$$ IN\ast C\ast IS=V $$

Value is broader than intervention effects alone. It reflects the optimization of a *configuration* of patient (Vpt), provider (Vpr), organization (Vo), and system (Vs) values and outcomes. Thus, value is a multicomponent, multilevel construct that represents the perceived or real benefit for each stakeholder and for stakeholders combined: a multilevel value proposition. For example, value for a service system may be increased population health, while for an organization, it may be optimized service delivery and decreased costs. Concurrently, a clinical professional may view value as being able to consider individual patient needs and outcomes, and patients may value their own improved functioning in daily life and/or clinical outcomes.

But what then is success of an EBI? By focusing on value, we suggest that implementation success can be defined as the ability to optimize value across the different levels and stakeholders. This perspective on implementation success aligns with recent definitions of sustainability, which highlight the ability to continuously deliver benefits as key part of the construct [74], with adaptations being a strategy to promote it [75]. The value equation proposes that effects on certain clinical outcomes are necessary but not sufficient. An EBI also needs to maximize value for individual providers, for the organization, and for the system. This shifts the focus on implementation from getting an EBI in place, to thinking about its value more broadly, and being more egalitarian in considering the needs of multiple stakeholders, including recently identified “bridging factors” to optimize implementation across context levels [76].

The equation, with its focus on value, also has implications for intervention developers. It implies that moving from designing interventions to maximize efficacy to designing interventions that maximize value, for multiple stake-holders. According to the Value Equation, the intervention that is most efficacious may not be the one that also provides the most value. A less complex intervention that can be delivered by less skilled staff and that requires less implementation resources (e.g., supervision, re-organization of care) may result in higher value than an intervention that stands little chance of being used in practice [26]. This is consistent with approaches to maximizing public health impact where a given EBI may have a smaller effect size, but if it is sustained and reaches more patients then even a small effect sizes can have significant public health impact [77].

It is in relation to the multidimensional value configuration that fidelity and adaptation should be considered. Sometimes, fidelity is a way to optimize value, sometimes it is adaptations, and often it is a combination. This also means that fidelity might optimize one outcome and adaptation another. Furthermore, the different types of outcomes may be valued differently by different stakeholders. In this, we acknowledge that different stakeholders’ definitions of value may differ. In fact, they may often be misaligned, such as when an organization is required by the system to provide a service to a sub-population that does not request it. We suggest that the better implementers are at acknowledging and addressing these value conflicts, the higher the likelihood for successful and sustained implementation. Community–academic partnerships may be one bridging factor that may facilitate this process [78] by engaging stakeholders in jointly considering system, organization, and patient needs, increasing their understanding of others agendas and encouraging a transparent negotiation of how to best address different needs. Techniques such as concept mapping and co-created program logic (COP) may be useful to promote an understanding of divergent viewpoints, and an effective dialogue [79, 80].

Similarly, by moving from focus only on treatment effect to a value configuration, we can reconcile arguments for fidelity and adaptation in relation to equity. We simply propose focusing on equity of the value achieved by the equation as a whole (i.e., for all stakeholders across levels) rather than only equity in relation to the intervention.

### Transparency over all the value equation terms

One of the main arguments for fidelity is related to transparency: Fidelity to an EBI is needed for comparisons, accumulation of knowledge, and accountability. Our third proposition is that what is essential is *transparent* use. Thus, replication and accumulation of knowledge is still possible, but redefined to focus on transparency in relation to *all* terms in the Value Equation. In this, the Value Equation is consistent with recent calls for redefining replicability in clinical science (e.g., [81]). Requirements from funders to provide information on all equation terms would be helpful to push the development in this direction.

This proposition is consistent with calls for rigorous strategies to monitor, guide and evaluate fidelity [82–84] as well as adaptation, as increasingly has been acknowledged (e.g., [17, 26, 29, 31, 85, 86]). The Value Equation adds to this by proposing transparent reporting of all equation terms, and justification of fidelity and adaptation based on how it promotes fit between the EBI and context and in relation to how it impacts value. In this way, users will be supported in assessing INfi and INfc in subsequent implementations. Otherwise, the risk is what can be called “adaptation neglect,” a syndrome where adaptations pass unnoticed or undocumented regardless of how obvious they are.

### Toward personalized value equations

One of the main argument for fidelity is to enable accumulation of knowledge through replication, putting focus on only one of the terms of the equation. The Value Equation and the transparency proposition instead focuses on all terms, thereby facilitating a gradual increase in the precision of the knowledge of what works for whom and when (i.e., specificity) [87]. This requires sophisticated processes and infrastructure. One way to achieve this may be to create databases of the different ways in which an intervention has been used, in what context, and to what effect [26, 86, 88]. Such data, thus, can form the basis for a gradual increased understanding of what creates value for whom and shows how the logic of the Value Equation can look in practice. For example, in the Paediatric Oncology Department of Karolinska University Hospital in Sweden, when a child does not respond as expected to a treatment protocol, adaptations are made, and both adaptations and effects are documented. Data from similar cases are accumulated, creating additional arms in the ongoing comparative trial. In this way, data on intervention*context configurations are collected.

Nevertheless, building databases that reflect the whole Value Equation may increase the administrative burden on clinical staff and organizations as a whole. A way to circumvent this risk may be to build a data infrastructure where all stakeholders involved in the healthcare process (patients, providers, organizations, and system) are invited to share and use data for their specific needs, so that those entering the data also benefit from it in their daily operations [89]. Although such a development may seem utopian in many fragmented systems, there are examples of these learning healthcare systems, for instance, at Cincinnati Children’s Hospital Medical Center [89] and in rheumatology care in Sweden [90]. Researchers may, for example, use the data for comparative effectiveness studies, and healthcare system representatives for benchmarking. However, the most transformative aspect may be when patients and providers can use the system at the point of care to track how an EBI is used (fidelity and adaptation) and what value it creates for the specific patient. This is consistent with recent applications of measurement-based care, where data related to intervention, context and implementation is assessed real-time along with clinical data to guide clinical decision making [91].

Used in this way, the learning healthcare system [92] will provide the most precise version of “what works for whom, when” we can think of: personalized value equations in patient- or provider-driven *n* = 1 studies [93]. Aggregation of all *n* = 1 studies will then provide the basis for accumulation of knowledge of “what works for whom, when,” thereby bridging personalized medicine and the ideas for systemizing knowledge about adaptations as outlined in the Adaptome [26].

## Conclusions

In mathematics and statistics, we are used to thinking about how the different terms of an equation together determine the outcome. Implementation scientists can use the same approach to understand the product of an EBI, minding the context in which it is used and given the implementation strategies applied. The Value Equation is a theoretical proposition that reconciles the role of adaptation and fidelity in the research-to-practice pathway. The Value Equation states that the optimal value configuration of the intervention that can be obtained (V) is a product of the intervention (IN), the nature of the context (C) in which the intervention is being implemented, and how well the implementation strategy (IS) optimizes the intervention and the context. Fidelity and adaptation determine how these terms are mixed and, in turn, the end product: the value configuration it produces for multiple stakeholders.

The Value Equation contains three central propositions: 1) it positions implementation strategies as a way to create fit between EBIs and context, 2) it explicates that the product of implementation effort should move from emphasizing effects to emphasizing optimization of a multilevel value configuration, and 3) it shifts focus from fidelity to transparency over all terms of the equation. While there are many complexities in each of these propositions and in each of the terms in the equation, we suggest that the Value Equation be used to develop and test hypotheses that ultimately can help implementation science move toward a more granular understanding of how methods to promote the uptake of research findings can be optimized.

## Data Availability

NA

## Abbrevations

EBI/EBIs: Evidence-based intervention(s)
V: Value
Vs: value and fit of intervention in the system context
Vo: Value and fit of intervention in organizational context
Vpr: Value and fit of the intervention for the provider
Vpt: Value and fit of the intervention for the patient
IN: Intervention
INf: Extent to which the intervention is carried out as it was described (fidelity)
INfc: Fidelity-consistent adaptations
INfi: Fidelity-inconsistent adaptations
C: Context
Cs: System context
Co: Organizational context
Cpr: Provider context (e.g., professional discipline, training, attitudes toward the EBI)
Cpt: Patient context (e.g., target group)
IS: Implementation Strategy
ISc: Implementation strategy optimizing the context
ISi: Implementation strategy optimizing the intervention
